# Heated Tobacco Product Smokers in Japan Identified by a Population-Based Survey

**DOI:** 10.2188/jea.JE20190199

**Published:** 2020-12-05

**Authors:** Aya Kinjo, Yuki Kuwabara, Maya Fujii, Aya Imamoto, Yoneatsu Osaki, Ruriko Minobe, Hitoshi Maezato, Hideaki Nakayama, Tsuyoshi Takimura, Susumu Higuchi

**Affiliations:** 1 Division of Environmental and Preventive Medicine, Department of Social Medicine, Faculty of Medicine, Tottori University, Tottori, Japan; 2 National Hospital Organization Kurihama Medical and Addiction Center, Kanagawa, Japan

**Keywords:** heated tobacco products, heat-not-burn tobacco, tobacco, population-based study, Japan

## Abstract

**Background:**

In this study, we aim to estimate the prevalence of heated tobacco product (HTP) smokers 3 years after the launch of HTPs in Japan.

**Methods:**

Our study, performed in February 2018 in Japan, had a cross-sectional population-based design. A total of 4,628 adult participants (2,121 men and 2,507 women) were randomly sampled from all regions of Japan. The response rate was 57.9%. Interviews were conducted by trained investigators who visited participants’ homes. A survey on current (past 30 days) and lifetime tobacco use (including e-cigarettes and HTPs), as well as numerous sociodemographic factors, was conducted.

**Results:**

The age-adjusted rates and estimated number of lifetime-HTP smokers were 14.1% (95% confidence interval [CI], 12.5–15.6%; 7.11 million men) and 3.7% (95% CI, 2.9–4.4%; 1.99 million women). The age-adjusted rates for current HTP smokers were 8.3% (95% CI, 7.1–9.6%; 4.21 million men) and 1.9% (95% CI, 1.3–2.4%; 1.02 million women). Multiple variables were found to be associated with a higher prevalence of current HTP use, including being male, aged 20–39 years, a current Internet user, a risky drinker, or a heavy episodic drinker. HTP use was also higher among men with 10 years or more of education, women with 15 years or less of education, and men with middle- or high-level household incomes.

**Conclusion:**

We concluded that HTP use has increased substantially in Japan. However, regulations for HTPs are weaker than those for combustible cigarettes in Japan. Thus, HTPs should be subjected to the same regulations as combustible tobacco products.

## INTRODUCTION

Heated tobacco products (HTPs) are relatively new, sold by several tobacco companies, and are used with electronic devices that, without combustion, allow smokers to inhale aerosols produced by heated tobacco leaves.^1^^–^^3^ Tobacco companies advertise HTPs as being relatively less harmful than other forms of tobacco, yet HTP aerosols contain nicotine and other chemicals,^4^^–^^6^ and the potential harm of secondhand exposure to HTPs has been reported.^7^^,^^8^ In 2014, the first HTP, IQOS, was created in Japan. By 2018, the IQOS market share of tobacco sales in Japan reached 15.5%.^9^ Thus, it is necessary to understand the present increase in HTP use from a public health perspective by conducting an empirical investigation into potential harmful effects of HTPs. Given their efficient implementation, Internet-based surveys have predominantly been used to provide current estimates of the prevalence of HTP use in three countries.^8^^,^^10^^,^^11^ However, to the best of our knowledge, no population-based studies on HTP prevalence have yet been reported. The participants sampled in web-based surveys may be potentially biased in terms of age and high familiarity with information technology, as participants are typically recruited from a voluntary registered pool. Therefore, the current study was conducted to provide a more accurate estimate of HTP users in Japan by utilizing a nationwide population-based survey.

## METHODS

### Design

In this cross-sectional study, adults were randomly sampled from all regions of Japan. The respondents were visited at home and interviewed by trained investigators.

### Participants

Participants were recruited using a stratified, two-stage random sampling approach. The strata were determined by first dividing the survey districts into eleven areas (Hokkaido, Tohoku, Kanto, Hokuriku, Tosan [Yamanashi, Nagano, and Gifu], Tokai, Kinki, Chugoku, Shikoku, Northern Kyushu, and Southern Kyushu) and then into five groups classified by municipality size (large cities, *n* = 14; cities with populations ≥300,000, ≥100,000, <100,000, and smaller towns and villages). The survey districts were selected from each stratum in proportion to the adult (≥20 years old) population. Survey data were collected from participants during the years of 2003 (*n* = 3,500), 2008 (*n* = 7,500), 2013 (*n* = 7,500), and 2018 (*n* = 8,000). This study utilized the 2018 survey dataset, as it included questions on HTPs. The datasets from 2003, 2008, and 2013 were utilized to calculate percentages of tobacco users.

### Survey procedures and response rates

The 2018 survey was conducted in February and March of that year. A survey request document was sent to the municipal office after the surveying district was randomly selected. Participants were then randomly selected by the investigator from the resident register at the municipal office. To ensure that participants provided informed consent, they were asked whether or not they would participate in the survey and, if they voluntarily agreed, the investigator visited their residence and conducted the interview. The number of participants and response rate were 4,628 and 57.9%, respectively. The details of previous surveys have been reported.^12^

### Indicators of tobacco use

The question on general tobacco use was, “Have you smoked any tobacco often or daily within the past 30 days? (Yes/No)”. The following question on HTPs was answered by any current (ie, past 30 days) tobacco user: “Have you ever used heated tobacco products such as iQOS, glo, or Ploom TECH?”. The question regarding e-cigarettes asked: “Have you ever used e-cigarettes such as FLEVO, EMILI, VITAFUL, or VITACIG?”. The answer choices for those two questions were: “never used”, “have used before, but not within the past 30 days”, and “currently use”. These items identified “any tobacco smoker, past 30 days”, “HTP smoker, lifetime”, “HTP smoker past 30 days”, “e-cigarette smoker, lifetime”, and “e-cigarette smoker, past 30 days”. In addition, participants were categorized into four groups: “non-smoker”, “only smokes combustible tobacco”, “HTP smoker or dual smoker”, and “other”.

### Socio-demographic, Internet use, and alcohol use indicators

Participants were classified into the following age groups: 20–29 years, 30–39 years, 40–49 years, 50–59 years, 60–69 years, 70–79 years, and 80 years and older. Educational attainment was classified into four categories: ≤9 years of education (junior high school level), 10–12 years of education (senior high school level), 13–15 years of education (technical school level or current university students), and ≥16 years of education (university and graduate school level). Marital status was classified into three categories: married or living with a partner, bereaved or divorced, and unmarried. Household size was classified into three categories: living alone, two persons, and three persons or more. Working status was classified according to six categories: regular employee, self-employed, non-regular employee, student, housework, and unemployed. There was no housework status classification for men. Household income was classified into three categories: <4,000,000 yen per year, 4,000,000–8,000,000 yen per year, and ≥8,000,000 yen per year (100 yen = 0.92 dollars as of February 1, 2018). In 2016, the median and average household incomes in Japan were approximately 4,420,000 and 5,602,000 yen, respectively.^13^ Individuals who used the Internet within the past 30 days were defined as current Internet users. Risky drinkers were defined by daily alcohol consumption (≥40 g for men or ≥20 g for women), which were the levels adopted in the second term of the National Health Promotion Movement of the 21^st^ century (Health Japan 21).^14^ Heavy episodic drinking was defined as drinking ≥60 g of alcohol on a single occasion within the past 30 days.^15^

### Statistical analyses

The age-adjusted rates and estimated numbers of HTP users were weighted based on the population of Japan in October 1, 2017. To calculate lower and upper 95% confidence intervals (CIs) regarding any tobacco, HTP, and e-cigarette smokers, the following formula was used: age-adjusted point estimate ± 1.96 × standard error of age-adjusted rate. Regarding the proportion of any tobacco, HTP, and e-cigarette users by sociodemographic background, crude rates were used, and 95% CIs were calculated, without adjusting for age. Individuals who did not respond to questions were included in the analysis as non-respondents. Statistical analyses were performed using Microsoft Excel 2016 software for Windows (Microsoft Corp., Redmond, WA, USA).

### Ethical considerations

The study protocol was approved by the ethics committee at the Kurihama Medical and Addiction Centre. During the visit for the interview, the investigator obtained informed consent from participants after providing a comprehensive explanation of the purpose of the investigation, its content, and how personal information would be protected. Researchers did not collect any personally identifiable information from the respondents, as it was excluded from the survey data.

## RESULTS

A total of 2,121 men and 2,507 women participated in the 2018 nationwide survey. Participant characteristics are shown in Table 1. Almost 70% of participants were current Internet users. The prevalence of any current tobacco use had steadily decreased between 2003 and 2013, but plateaued between 2013 and 2018.

**Table 1.** Participant characteristics

	Men		Women		Total	
		
	*n*	(%)	*N*	(%)	*n*	(%)
Total	2,121	45.8	2,507	54.2	4,628	100.0

Age groups, years						
20–29	167	7.9	197	7.9	364	7.9
30–39	262	12.4	316	12.6	578	12.5
40–49	362	17.1	476	19.0	838	18.1
50–59	310	14.6	398	15.9	708	15.3
60–69	426	20.1	490	19.5	916	19.8
70–79	422	19.9	411	16.4	833	18.0
80 years and older	172	8.1	219	8.7	391	8.4

Areas						
Hokkaido	103	4.9	109	4.3	212	4.6
Tohoku	182	8.6	193	7.7	375	8.1
Kanto	623	29.4	751	30.0	1,374	29.7
Hokuriku	103	4.9	130	5.2	233	5.0
Tosan	107	5.0	109	4.3	216	4.7
Tokai	242	11.4	257	10.3	499	10.8
Kinki	326	15.4	388	15.5	714	15.4
Chugoku	124	5.8	179	7.1	303	6.5
Shikoku	67	3.2	77	3.1	144	3.1
Northern Kyushu	135	6.4	169	6.7	304	6.6
Southern Kyushu	109	5.1	145	5.8	254	5.5

Municipality size						
Large cities	512	24.1	609	24.3	1,121	24.2
Cities with populations ≥300,000	349	16.5	416	16.6	765	16.5
Cities with populations ≥100,000	544	25.6	679	27.1	1,223	26.4
Cities with populations <100,000	507	23.9	564	22.5	1,071	23.1
Smaller towns and villages	209	9.9	239	9.5	448	9.7

Educational attainment						
1–9 years	232	10.9	297	11.8	529	11.4
10–12 years	797	37.6	1,092	43.6	1,889	40.8
13–15 years	289	13.6	681	27.2	970	21
16 years	798	37.6	435	17.4	1,233	26.6
No response	5	0.2	2	0.1	7	0.2

Marital status						
Married	1,573	74.2	1,730	69.0	3,303	71.4
Bereaved or divorced	139	6.6	433	17.3	572	12.4
Unmarried	404	19.0	339	13.5	743	16.1
No response	5	0.2	5	0.2	10	0.2

Number of cohabitants						
Alone	202	9.5	246	9.8	448	9.7
2 persons	695	32.8	703	28.0	1,398	30.2
3 or more persons	1,221	57.6	1,558	62.2	2,779	60.0
No response	3	0.1	0	0.0	3	0.1

Working status						
Employee (regular)	939	44.3	477	19.0	1,416	30.6
Employee (non-regular)	218	10.3	670	26.7	888	19.2
Self-employed	296	14.0	195	7.8	491	10.6
Student	40	1.9	37	1.5	77	1.7
Housework	0	0.0	865	34.5	865	18.7
Unemployed	624	29.4	256	10.2	880	19.0
Others	4	0.2	7	0.3	11	0.2

Annual household income						
<4,000,000 yen	732	34.5	819	32.7	1,551	33.5
4,000,000–8,000,000 yen	652	30.7	593	23.7	1,245	26.9
≥8,000,000 yen	368	17.4	389	15.5	757	16.4
No response	369	17.4	706	28.2	1,075	23.2
Internet user, past 30 days	1,499	70.7	1,632	65.1	3,131	67.7

Risky drinker (male 40 g/day, female 20 g/day or more)	316	14.9	169	6.7	485	10.5

Heavy episodic drinker, past 30 days	649	30.6	205	8.2	854	18.5

Any tobacco use, past 30 days						
Year 2003	555	46.9	198	14.5	753	29.6
Year 2008	761	40.5	258	11.5	1,019	24.7
Year 2013	570	30.5	206	9.0	776	18.7
Year 2018	637	30.0	242	9.4	879	19.0

Use of new tobacco products						
HTP^a^ smoker, lifetime (Year 2018)	264	12.4	90	3.6	354	7.6
HTP^a^ smoker, past 30 days (Year 2018)	131	7.3	55	1.8	186	4.3
E-cigarette smoker, lifetime (Year 2018)	155	6.2	45	2.2	200	4.0
E-cigarette smoker, past 30 days (Year 2018)	31	1.5	11	0.4	42	0.9

Smoking patterns						
Non-smoker	1,484	70.0	2,265	90.3	3,749	81.0
Only smoke combustible tobacco	473	22.3	197	7.9	670	14.5
HTP^a^ smoker or dual smoker	133	6.3	34	1.4	167	3.6
Other	31	1.4	11	0.4	42	0.9

^a^HTP, heated tobacco products.

**Figure 1.**
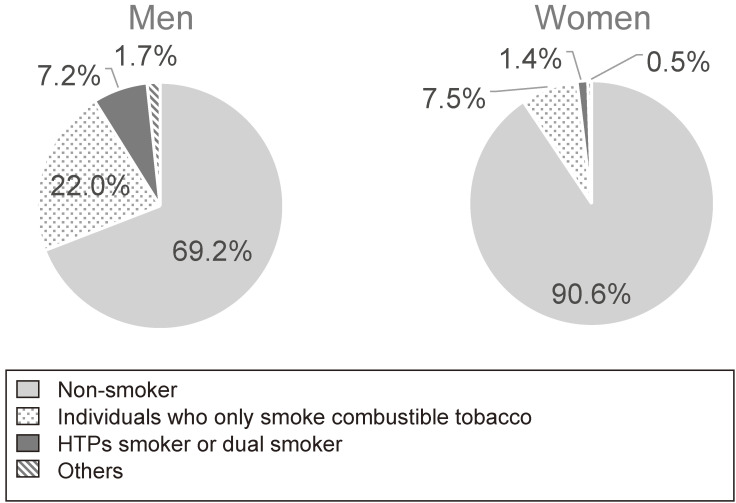
Proportions of tobacco smoking habits in Japan

Table 2 shows the estimates of various tobacco product smokers. The age-adjusted rate and estimated number of current HTP smokers in the Japanese population was 5.23 million, with 4.21 million men (8.3%; 95% CI, 7.1–9.6%) and 1.02 million women (1.9%; 95% CI, 1.3–2.4%). The age-adjusted rate of current users of any type of tobacco was 30.8% (95% CI, 28.8–32.8%) among men and 9.4% (95% CI, 8.3–10.5%) among women. Almost one-third of men and one-fifth of women were HTP smokers in the tobacco-user population. The age-adjusted rate of current e-cigarette smokers was 1.6% (95% CI, 1.0–2.2%) among men and 0.5% (95% CI, 0.2–0.7%) among women, and the number of HTP smokers was higher than e-cigarette smokers. The age adjusted rate estimates were calculated for “non-smoker” (men = 69.2%; women = 90.6%), “only smoke combustible tobacco” (men = 22.0%; women = 7.5%), “HTP smoker or dual smoker” (men = 7.2%; women = 1.4%), and “other” (men = 1.7%; women = 0.5%) groups (see Figure 1).

**Table 2.** Frequency and estimates of tobacco product use

		Men	Women	Total
Any tobacco use, past 30 days	Crude rate (%)	30.0	9.7	19.0
	Age-adjusted rate^a^ (%)	30.8	9.4	19.7
	95% CI of the proportion (%)	28.8–32.8	8.3–10.5	18.6–20.8
	Estimated number (in millions)	15.57	5.13	20.70
	95% CI of the estimated number (in millions)	14.56–16.58	4.51–5.75	19.52–21.89

HTP smoker, lifetime	Crude rate (%)	12.4	3.6	7.6
	Age-adjusted rate^a^ (%)	14.1	3.7	8.7
	95% CI of the proportion (%)	12.5–15.6	2.9–4.4	7.8–9.5
	Estimated number (in millions)	7.11	1.99	9.10
	95% CI of the estimated number (in millions)	6.32–7.90	1.59–2.40	8.21–9.99

HTP smoker, past 30 days	Crude rate (%)	7.3	1.8	4.3
	Age-adjusted rate^a^ (%)	8.3	1.9	5.0
	95% CI of the proportion (%)	7.1–9.6	1.3–2.4	4.3–5.6
	Estimated number (in millions)	4.21	1.02	5.23
	95% CI of the estimated number (in millions)	3.58–4.84	0.73–1.32	4.54–5.93

E-cigarette smoker, lifetime	Crude rate (%)	6.2	2.2	4.0
	Age-adjusted rate^a^ (%)	6.8	2.3	4.4
	95% CI of the proportion (%)	5.6–7.9	1.7–2.9	3.8–5.1
	Estimated number (in millions)	3.42	1.23	4.65
	95% CI of the estimated number (in millions)	2.84–4.00	0.91–1.56	3.99–5.32

E-cigarette smoker, past 30 days	Crude rate (%)	1.5	0.4	0.9
	Age-adjusted rate^a^ (%)	1.6	0.5	1.0
	95% CI of the proportion (%)	1.0–2.2	0.2–0.7	0.7–1.3
	Estimated number (in millions)	0.82	0.25	1.07
	95% CI of the estimated number (in millions)	0.53–1.11	0.10–0.40	0.74–1.40

CI, confidence interval. HTP, heated tobacco products.

^a^The 2017 population data vital statistics were used to adjust for age.

Table 3 shows the crude rate of various types of tobacco smokers among men by sociodemographic factors. The prevalence of HTP smokers was highest among the following groups: 20–49-year-olds, residents of Tosan, individuals with 10 years or more of education, individuals who were not bereaved or divorced, individuals with 4,000,000 yen or more annual household income, current Internet users, risky drinkers, and heavy episodic drinkers.

**Table 3.** Male tobacco smoker types by sociodemographic characteristics

	Any tobacco use, past 30 days		HTP smoker, lifetime		HTP smoker, past 30 days		E-cigarette smoker, lifetime		E-cigarette smoker, past 30 days	
				
	%	95% CI (%)	%	95% CI (%)	%	95% Cl (%)	%	95% CI (%)	%	95% CI (%)
Total	30.0	(28.0–32.0)	12.4	(11.0–13.8)	7.3	(6.2–8.4)	6.2	(5.2–7.2)	1.5	(1.0–2.0)

Age groups, years										
20–29	26.3	(24.4–28.2)	22.2	(20.4–24.0)	12.6	(11.2–14.0)	10.8	(9.5–12.1)	2.4	(1.7–3.1)
30–39	31.7	(29.7–33.7)	20.6	(18.9–22.3)	13.4	(12.0–14.8)	9.9	(8.6–11.2)	3.1	(2.4–3.8)
40–49	41.7	(39.6–43.8)	21.0	(19.3–22.7)	13.3	(11.9–14.7)	7.2	(6.1–8.3)	1.7	(1.1–2.3)
50–59	37.4	(35.3–39.5)	14.2	(12.7–15.7)	7.7	(6.6–8.8)	7.1	(6.0–8.2)	1.3	(0.8–1.8)
60–69	32.6	(30.6–34.6)	8.9	(7.7–10.1)	5.6	(4.6–6.6)	5.2	(4.3–6.1)	1.9	(1.3–2.5)
70–79	20.6	(18.9–22.3)	3.1	(2.4–3.8)	0.7	(0.3–1.1)	3.3	(2.5–4.1)	0.2	(0.0–0.4)
80 years and elder	9.9	(8.6–11.2)	1.2	(0.7–1.7)	0.0	(0.0–0.0)	1.7	(1.1–2.3)	0.0	(0.0–0.0)

Area										
Hokkaido	32.0	(30.0–34.0)	10.7	(9.4–12.0)	5.8	(4.8–6.8)	10.7	(9.4–12.0)	4.9	(4.0–5.8)
Tohoku	31.9	(29.9–33.9)	10.4	(9.1–11.7)	8.2	(7.0–9.4)	3.8	(3.0–4.6)	1.1	(0.7–1.5)
Kanto	28.4	(26.5–30.3)	12.4	(11.0–13.8)	6.7	(5.6–7.8)	6.3	(5.3–7.3)	1.3	(0.8–1.8)
Hokuriku	36.9	(34.8–39.0)	16.5	(14.9–18.1)	9.7	(8.4–11.0)	11.7	(10.3–13.1)	2.9	(2.2–3.6)
Tosan	38.3	(36.2–40.4)	20.6	(18.9–22.3)	15.9	(14.3–17.5)	9.3	(8.1–10.5)	3.7	(2.9–4.5)
Tokai	31.8	(29.8–33.8)	13.6	(12.1–15.1)	8.3	(7.1–9.5)	5.0	(4.1–5.9)	0.4	(0.1–0.7)
Kinki	26.4	(24.5–28.3)	12.3	(10.9–13.7)	8.3	(7.1–9.5)	4.3	(3.4–5.2)	1.5	(1.0–2.0)
Chugoku	25.8	(23.9–27.7)	8.9	(7.7–10.1)	3.2	(2.5–3.9)	4.0	(3.2–4.8)	0.8	(0.4–1.2)
Shikoku	20.9	(19.2–22.6)	7.5	(6.4–8.6)	3.0	(2.3–3.7)	4.5	(3.6–5.4)	0.0	(0.0–0.0)
Northern Kyushu	32.6	(30.6–34.6)	12.6	(11.2–14.0)	5.2	(4.3–6.1)	5.9	(4.9–6.9)	0.0	(0.0–0.0)
Southern Kyushu	33.9	(31.9–35.9)	11.0	(9.7–12.3)	4.6	(3.7–5.5)	9.2	(8.0–10.4)	1.8	(1.2–2.4)

Municipality size										
Large cities	25.4	(23.5–27.3)	11.1	(9.8–12.4)	7.0	(5.9–8.1)	6.1	(5.1–7.1)	1.6	(1.1–2.1)
Cities with populations ≥300,000	28.1	(26.2–30.0)	12.0	(10.6–13.4)	6.3	(5.3–7.3)	6.0	(5.0–7.0)	1.4	(0.9–1.9)
Cities with populations ≥100,000	32.2	(30.2–34.2)	12.9	(11.5–14.3)	7.2	(6.1–8.3)	5.7	(4.7–6.7)	1.3	(0.8–1.8)
Cities with populations <100,000	33.1	(31.1–35.1)	14.0	(12.5–15.5)	8.7	(7.5–9.9)	6.5	(5.5–7.5)	1.4	(0.9–1.9)
Smaller towns and villages	31.6	(29.6–33.6)	11.5	(10.1–12.9)	6.7	(5.6–7.8)	7.2	(6.1–8.3)	1.9	(1.3–2.5)

Educational attainment										
1–9 years	29.3	(27.4–31.2)	6.9	(5.8–8.0)	3.9	(3.1–4.7)	3.9	(3.1–4.7)	0.4	(0.1–0.7)
10–12 years	35.0	(33.0–37.0)	13.6	(12.1–15.1)	7.9	(6.8–9.0)	8.0	(6.8–9.2)	1.8	(1.2–2.4)
13–15 years	31.5	(29.5–33.5)	15.6	(14.1–17.1)	9.3	(8.1–10.5)	7.6	(6.5–8.7)	1.7	(1.1–2.3)
≥16 years	24.7	(22.9–26.5)	11.9	(10.5–13.3)	7.0	(5.9–8.1)	4.5	(3.6–5.4)	1.4	(0.9–1.9)
No response	40.0	(37.9–42.1)	0.0	(0.0–0.0)	0.0	(0.0–0.0)	0.0	(0.0–0.0)	0.0	(0.0–0.0)

Marital status										
Married	28.8	(26.9–30.7)	12.0	(10.6–13.4)	7.2	(6.1–8.3)	5.5	(4.5–6.5)	1.4	(0.9–1.9)
Bereaved or divorced	39.6	(37.5–41.7)	9.4	(8.2–10.6)	5.0	(4.1–5.9)	7.2	(6.1–8.3)	2.2	(1.6–2.8)
Unmarried	31.7	(29.7–33.7)	15.6	(14.1–17.1)	8.7	(7.5–9.9)	8.7	(7.5–9.9)	1.5	(1.0–2.0)
No response	20.0	(18.3–21.7)	0.0	(0.0–0.0)	0.0	(0.0–0.0)	0.0	(0.0–0.0)	0.0	(0.0–0.0)

Number of cohabitants										
Alone	35.1	(33.1–37.1)	12.4	(11.0–13.8)	5.9	(4.9–6.9)	8.9	(7.7–10.1)	1.5	(1.0–2.0)
2 persons	25.2	(23.4–27.0)	7.9	(6.8–9.0)	5.5	(4.5–6.5)	4.3	(3.4–5.2)	1.4	(0.9–1.9)
3 or more persons	31.9	(29.9–33.9)	15.1	(13.6–16.6)	8.6	(7.4–9.8)	6.8	(5.7–7.9)	1.5	(1.0–2.0)
No response	66.7	(64.7–68.7)	0.0	(0.0–0.0)	0.0	(0.0–0.0)	0.0	(0.0–0.0)	0.0	(0.0–0.0)

Working status										
Employee (regular)	35.6	(33.6–37.6)	18.3	(16.7–19.9)	11.4	(10.0–12.8)	8.3	(7.1–9.5)	2.3	(1.7–2.9)
Employee (non-regular)	33.5	(31.5–35.5)	11.5	(10.1–12.9)	6.9	(5.8–8.0)	6.4	(5.4–7.4)	0.5	(0.2–0.8)
Self-employed	31.4	(29.4–33.4)	13.9	(12.4–15.4)	6.1	(5.1–7.1)	7.1	(6.0–8.2)	1.0	(0.6–1.4)
Student	17.5	(15.9–19.1)	15.0	(13.5–16.5)	12.5	(11.1–13.9)	7.5	(6.4–8.6)	5.0	(4.1–5.9)

Housework										
Unemployed	20.8	(19.1–22.5)	3.2	(2.5–3.9)	1.6	(1.1–2.1)	2.4	(1.7–3.1)	0.5	(0.2–0.8)
Other	0.0	(0.0–0.0)	0.0	(0.0–0.0)	0.0	(0.0–0.0)	0.0	(0.0–0.0)	0.0	(0.0–0.0)

Annual household income										
<4,000,000 yen	29.8	(27.9–31.7)	9.0	(7.8–10.2)	4.4	(3.5–5.3)	5.2	(4.3–6.1)	1.1	(0.7–1.5)
4,000,000–8,000,000 yen	36.3	(34.3–38.3)	17.0	(15.4–18.6)	11.3	(10.0–12.6)	7.7	(6.6–8.8)	2.0	(1.4–2.6)
≥8,000,000 yen	25.5	(23.6–27.4)	14.4	(12.9–15.9)	8.4	(7.2–9.6)	6.8	(5.7–7.9)	1.1	(0.7–1.5)
No response	23.8	(22.0–25.6)	9.2	(8.0–10.4)	4.9	(4.0–5.8)	4.9	(4.0–5.8)	1.6	(1.1–2.1)

Internet use, past 30 days										
Non-Internet user, past 30 days	27.3	(25.4–29.2)	3.7	(2.9–4.5)	1.1	(0.7–1.5)	3.4	(2.6–4.2)	0.5	(0.2–0.8)
Internet user, past 30 days	31.2	(29.2–33.2)	16.1	(14.5–17.7)	9.9	(8.6–11.2)	7.3	(6.2–8.4)	1.9	(1.3–2.5)

Risky drinking										
Drink alcohol less than 40 g/day	28.1	(26.2–30.0)	11.6	(10.2–13.0)	6.8	(5.7–7.9)	5.9	(4.9–6.9)	1.4	(0.9–1.9)
Drink alcohol more than 40 g/day	40.8	(38.7–42.9)	17.4	(15.8–19.0)	10.1	(8.8–11.4)	7.9	(6.8–9.0)	1.6	(1.1–2.1)

Heavy Episodic drinking										
Non-heavy episodic drinker, past 30 days	27.6	(25.7–29.5)	9.6	(8.3–10.9)	5.5	(4.5–6.5)	5.0	(4.1–5.9)	1.0	(0.6–1.4)
Heavy episodic drinker, past 30 days	35.6	(33.6–37.6)	19.0	(17.3–20.7)	11.4	(10.0–12.8)	8.9	(7.7–10.1)	2.5	(1.8–3.2)

Table 4 shows the crude rate of various tobacco smoker types among women by sociodemographic factors. The prevalence of HTP smokers was highest in the following groups: 20–39-year-olds, individuals with 15 years or less of education, individuals with a household size of three or more persons, individuals who were employed or self-employed, current Internet users, risky drinkers, and heavy episodic drinkers.

**Table 4.** Female tobacco smoker types by sociodemographic characteristics

	Any tobacco use, past 30 days		HTP smoker, lifetime		HTP smoker, past 30 days		E-cigarette smoker, lifetime		E-cigarette smoker, past 30 days	
				
	%	95% CI (%)	%	95% CI (%)	%	95% CI (%)	%	95% CI (%)	%	95% CI (%)
Total	9.7	(8.5–10.9)	3.6	(2.9–4.3)	1.8	(1.3–2.3)	2.2	(1.6–2.8)	0.4	(0.2–0.6)

Age groups, years										
20–29	8.6	(7.5–9.7)	6.1	(5.2–7.0)	4.1	(3.3–4.9)	4.1	(3.3–4.9)	0.5	(0.2–0.8)
30–39	13.6	(12.3–14.9)	7.9	(6.8–9.0)	4.7	(3.9–5.5)	5.4	(4.5–6.3)	1.9	(1.4–2.4)
40–49	12.4	(11.1–13.7)	5.0	(4.1–5.9)	2.5	(1.9–3.1)	2.3	(1.7–2.9)	0.8	(0.5–1.1)
50–59	13.8	(12.4–15.2)	4.3	(3.5–5.1)	2.3	(1.7–2.9)	2.5	(1.9–3.1)	0.0	(0.0–0.0)
60–69	9.6	(8.4–10.8)	2.0	(1.5–2.5)	0.2	(0.0–0.4)	1.6	(1.1–2.1)	0.0	(0.0–0.0)
70–79	3.9	(3.1–4.7)	0.2	(0.0–0.4)	0.0	(0.0–0.0)	0.2	(0.0–0.4)	0.0	(0.0–0.0)
80 years and elder	2.3	(1.7–2.9)	0.5	(0.2–0.8)	0.0	(0.0–0.0)	0.0	(0.0–0.0)	0.0	(0.0–0.0)

Area										
Hokkaido	13.8	(12.4–15.2)	1.8	(1.3–2.3)	0.9	(0.5–1.3)	0.9	(0.5–1.3)	0.0	(0.0–0.0)
Tohoku	10.9	(9.7–12.1)	5.2	(4.3–6.1)	2.1	(1.5–2.7)	3.1	(2.4–3.8)	0.5	(0.2–0.8)
Kanto	9.9	(8.7–11.1)	4.3	(3.5–5.1)	2.5	(1.9–3.1)	2.7	(2.1–3.3)	0.5	(0.2–0.8)
Hokuriku	6.2	(5.3–7.1)	3.1	(2.4–3.8)	2.3	(1.7–2.9)	0.0	(0.0–0.0)	0.0	(0.0–0.0)
Tosan	11.0	(9.8–12.2)	2.8	(2.2–3.4)	0.9	(0.5–1.3)	2.8	(2.2–3.4)	0.9	(0.5–1.3)
Tokai	8.2	(7.1–9.3)	3.5	(2.8–4.2)	3.1	(2.4–3.8)	2.3	(1.7–2.9)	0.8	(0.5–1.1)
Kinki	10.1	(8.9–11.3)	3.6	(2.9–4.3)	1.0	(0.6–1.4)	2.1	(1.5–2.7)	0.3	(0.1–0.5)
Chugoku	10.6	(9.4–11.8)	3.4	(2.7–4.1)	0.6	(0.3–0.9)	2.8	(2.2–3.4)	0.0	(0.0–0.0)
Shikoku	11.7	(10.4–13)	3.9	(3.1–4.7)	2.6	(2.0–3.2)	0.0	(0.0–0.0)	0.0	(0.0–0.0)
Northern Kyushu	11.2	(10.0–12.4)	1.8	(1.3–2.3)	0.6	(0.3–0.9)	1.8	(1.3–2.3)	0.6	(0.3–0.9)
Southern Kyushu	3.4	(2.7–4.1)	2.8	(2.2–3.4)	0.7	(0.4–1.0)	2.1	(1.5–2.7)	0.7	(0.4–1.0)

Municipality size										
Large cities	10.5	(9.3–11.7)	3.8	(3.1–4.5)	1.8	(1.3–2.3)	2.5	(1.9–3.1)	0.8	(0.5–1.1)
Cities with populations ≥300,000	6.0	(5.1–6.9)	1.7	(1.2–2.2)	1.0	(0.6–1.4)	1.4	(0.9–1.9)	0.5	(0.2–0.8)
Cities with populations ≥100,000	10.6	(9.4–11.8)	4.1	(3.3–4.9)	1.8	(1.3–2.3)	2.8	(2.2–3.4)	0.0	(0.0–0.0)
Cities with populations <100,000	10.6	(9.4–11.8)	4.4	(3.6–5.2)	2.5	(1.9–3.1)	2.3	(1.7–2.9)	0.5	(0.2–0.8)
Smaller towns and villages	8.8	(7.7–9.9)	2.9	(2.2–3.6)	1.7	(1.2–2.2)	0.8	(0.5–1.1)	0.4	(0.2–0.6)

Educational attainment										
1–9 years	10.4	(9.2–11.6)	2.7	(2.1–3.3)	1.7	(1.2–2.2)	2.0	(1.5–2.5)	0.7	(0.4–1.0)
10–12 years	11.6	(10.3–12.9)	4.0	(3.2–4.8)	1.8	(1.3–2.3)	2.4	(1.8–3.0)	0.5	(0.2–0.8)
13–15 years	10.0	(8.8–11.2)	5.1	(4.2–6.0)	2.6	(2.0–3.2)	3.4	(2.7–4.1)	0.6	(0.3–0.9)
≥16 years	3.7	(3.0–4.4)	0.7	(0.4–1.0)	0.5	(0.2–0.8)	0.0	(0.0–0.0)	0.0	(0.0–0.0)
No response	0.0	(0.0–0.0)	0.0	(0.0–0.0)	0.0	(0.0–0.0)	0.0	(0.0–0.0)	0.0	(0.0–0.0)

Marital status										
Married	8.4	(7.3–9.5)	3.5	(2.8–4.2)	2.0	(1.5–2.5)	2.0	(1.5–2.5)	0.5	(0.2–0.8)
Bereaved or divorced	13.6	(12.3–14.9)	3.5	(2.8–4.2)	0.5	(0.2–0.8)	2.8	(2.2–3.4)	0.0	(0.0–0.0)
Unmarried	10.9	(9.7–12.1)	4.4	(3.6–5.2)	2.7	(2.1–3.3)	2.7	(2.1–3.3)	0.9	(0.5–1.3)
No response	0.0	(0.0–0.0)	0.0	(0.0–0.0)	0.0	(0.0–0.0)	0.0	(0.0–0.0)	0.0	(0.0–0.0)

Number of cohabitants										
Alone	11.4	(10.2–12.6)	1.2	(0.8–1.6)	0.4	(0.2–0.6)	0.8	(0.5–1.1)	0.4	(0.2–0.6)
2 persons	10.4	(9.2–11.6)	2.8	(2.2–3.4)	1.1	(0.7–1.5)	1.7	(1.2–2.2)	0.0	(0.0–0.0)
3 or more persons	9.1	(8.0–10.2)	4.3	(3.5–5.1)	2.3	(1.7–2.9)	2.6	(2.0–3.2)	0.6	(0.3–0.9)
No response										

Working status										
Employee (regular)	12.4	(11.1–13.7)	5.5	(4.6–6.4)	2.5	(1.9–3.1)	2.7	(2.1–3.3)	0.4	(0.2–0.6)
Employee (non-regular)	13.3	(12.0–14.6)	5.1	(4.2–6.0)	2.2	(1.6–2.8)	3.6	(2.9–4.3)	0.7	(0.4–1.0)
Self-employed	12.3	(11.0–13.6)	5.1	(4.2–6.0)	3.1	(2.4–3.8)	3.1	(2.4–3.8)	1.0	(0.6–1.4)
Student	0.0	(0.0–0.0)	0.0	(0.0–0.0)	0.0	(0.0–0.0)	0.0	(0.0–0.0)	0.0	(0.0–0.0)
Housework	5.9	(5.0–6.8)	1.6	(1.1–2.1)	1.0	(0.6–1.4)	1.3	(0.9–1.7)	0.2	(0.0–0.4)
Unemployed	7.4	(6.4–8.4)	2.3	(1.7–2.9)	1.2	(0.8–1.6)	0.4	(0.2–0.6)	0.0	(0.0–0.0)
Other	0.0	(0.0–0.0)	0.0	(0.0–0.0)	0.0	(0.0–0.0)	0.0	(0.0–0.0)	0.0	(0.0–0.0)
Annual household income										
<4,000,000 yen	12.5	(11.2–13.8)	3.5	(2.8–4.2)	1.6	(1.1–2.1)	2.1	(1.5–2.7)	0.2	(0.0–0.4)
4,000,000–8,000,000 yen	9.8	(8.6–11.0)	4.7	(3.9–5.5)	2.5	(1.9–3.1)	2.2	(1.6–2.8)	0.8	(0.5–1.1)
≥8,000,000 yen	7.5	(6.5–8.5)	3.1	(2.4–3.8)	1.5	(1.0–2.0)	2.8	(2.2–3.4)	0.5	(0.2–0.8)
No response	7.5	(6.5–8.5)	3.0	(2.3–3.7)	1.6	(1.1–2.1)	2.0	(1.5–2.5)	0.3	(0.1–0.5)

Internet use, past 30 days										
Non-Internet user, past 30 days	7.2	(6.1–8.3)	0.9	(0.5–1.3)	0.1	(0.0–0.2)	1.0	(0.6–1.4)	0.1	(0.0–0.2)
Internet user, past 30 days	11.0	(9.7–12.3)	5.0	(4.1–5.9)	2.7	(2.0–3.4)	2.8	(2.1–3.5)	0.6	(0.3–0.9)

Risky drinking										
Drink alcohol less than 20 g/day	8.4	(7.3–9.5)	3.0	(2.3–3.7)	1.5	(1.0–2.0)	1.9	(1.4–2.4)	0.3	(0.1–0.5)
Drink alcohol more than 20 g/day	27.2	(25.5–28.9)	12.4	(11.1–13.7)	6.5	(5.5–7.5)	6.5	(5.5–7.5)	1.8	(1.3–2.3)

Heavy Episodic drinking										
Non-heavy episodic drinker, past 30 days	8.3	(7.2–9.4)	2.5	(1.9–3.1)	1.2	(0.8–1.6)	1.5	(1.0–2.0)	0.2	(0.0–0.4)
Heavy episodic drinker, past 30 days	25.4	(23.7–27.1)	15.6	(14.2–17.0)	8.8	(7.7–9.9)	10.2	(9.0–11.4)	2.9	(2.2–3.6)

## DISCUSSION

This is one of the first reports to estimate the prevalence HTP smokers using a national population-based survey. The current survey indicated that the estimated number of current HTP smokers in Japan was 4.21 million (8.3%) men and 1.02 million (1.9%) women, as of February 2018. The proportion of HTP smokers is more than one-fourth of the total tobacco-user population.

Several web-based studies have reported HTP prevalence. One study from Japan observed that the prevalence of IQOS use increased from 0.4% in 2015 to 10.6% in 2018 among men and from 0.2% in 2015 to 3.1% in 2018 among women.^8^^,^^16^ The 2018 follow-up survey was conducted at the same time as the current study. Considering the 2018 web-based survey was limited to IQOS, the current prevalence estimation is lower than that of the previous study. The difference could be explained by the differences in the age range of participants, the characteristics of participants between web-based surveys and face-to-face interviews, and the research design between cohort studies and cross-sectional studies.

The trend of a decline in tobacco use ceased between 2013 and 2018. While it is unclear whether the current plateau is associated with the launch of HTPs, tobacco industry marketing tactics that suggest HTPs are less harmful than traditional tobacco products may attract conscientious individuals concerned with their health.^17^ Additionally, HTPs are presented as sophisticated and clean, which may appeal to young individuals with no prior interest in tobacco.^17^^,^^18^ Thus, it is quite possible that the prevalence of tobacco use could have declined further if HTPs had not been introduced.

The present findings show that individuals living with three or more persons were more likely to be HTP smokers. This result may also be related to marketing campaigns from tobacco companies, as the harmful effects of tobacco smoke are well known in Japan, and smokers generally smoke outside their homes. As such, individuals concerned with second-hand smoke impacting family members might shift from cigarettes to HTPs. However, the harm incurred by HTPs cannot be ignored, and such forms of advertising by the tobacco industry arguably pose a health risk to users.^7^ The percentage of HTP users is higher among individuals with risky and/or heavy episodic drinking habits. Smoking habits and drinking habits are highly related, which is likely why HTPs are positively correlated with alcohol consumption.^19^ We also observed that the percentage of male HTP users was particularly high in Tosan, an area where the rate of combustible tobacco is also particularly high. However, the findings indicated that the highest percentage of female HTP users was in Tokai, the area where HTPs were first launched in Japan.

The current study has several limitations. The primary limitation is the likely bias associated with self-report measures. Biological samples were not provided by participants, and thus it is possible that participants provided inaccurate answers. To reduce response errors as much as possible, concise and easy questions were used. Additionally, the current survey was carried out in person, which may have reduced incorrect answers. The second limitation is that the sample size of each age group was too small to analyze age differences in HTP prevalence. The effect of age was strong among HTP smokers, and an age-stratified analysis should be conducted to examine related background factors. The fourth limitation is the cross-sectional study design, which does not allow for the verification of causal effects of sociodemographic and risk factors. However, the primary purpose of the current study to estimate the total percentage was not affected by this limitation. The methodology in the current study was suitable in terms of collecting highly representative samples.

In conclusion, the current survey indicated that the estimated number of current Japanese HTP smokers was 4.21 million (8.3%) men and 1.02 million (1.9%) women, as of February 2018. However, the regulations for HTPs in Japan is weaker than those for combustible cigarettes. As such, equivalent regulations should be extended to HTPs.
